# Immune response in ofatumumab treated multiple sclerosis patients after SARS-CoV-2 vaccination

**DOI:** 10.3389/fimmu.2022.980526

**Published:** 2022-08-31

**Authors:** Simon Faissner, Neele Heitmann, Carlos Plaza-Sirvent, Paulina Trendelenburg, Ulas Ceylan, Jeremias Motte, Clara Bessen, Doris Urlaub, Carsten Watzl, Oliver Overheu, Anke Reinacher-Schick, Kerstin Hellwig, Stephanie Pfaender, Ingo Schmitz, Ralf Gold

**Affiliations:** ^1^ Department of Neurology, Ruhr-University Bochum, St. Josef-Hospital, Bochum, Germany; ^2^ Department of Molecular Immunology, Ruhr-University Bochum, Bochum, Germany; ^3^ Department for Immunology, Leibniz Research Centre for Working Environment and Human Factors (IfADo) Technical University (TU) Dortmund, Dortmund, Germany; ^4^ Department of Hematology and Oncology with Palliative Care, St. Josef Hospital, Ruhr-University Bochum, Bochum, Germany; ^5^ Department of Molecular and Medical Virology, Ruhr-University Bochum, Bochum, Germany

**Keywords:** SARS-CoV-2 vaccination, multiple sclerosis, anti-CD20 therapy, T cell response, ofatumumab, humoral immune response

## Abstract

**Objective:**

The pandemic induced by SARS-CoV-2 has huge implications for patients with immunosuppression that is caused by disorders or specific treatments. Especially approaches targeting B cells *via* anti-CD20 therapy are associated with impaired humoral immune response but sustained cellular immunity. Ofatumumab is a human anti-CD20 directed antibody applied in low dosages subcutaneously, recently licensed for Multiple Sclerosis (MS). Effects of early ofatumumab treatment on alterations of immune cell composition and immune response towards SARS-CoV-2 are incompletely understood.

**Methods:**

We here investigated immune cell alterations in early ofatumumab (Ofa) treated patients and effects on humoral (titer, neutralization capacity against wild type, Delta and Omicron) and cellular immune responses in Ofa treated MS patients following a third vaccination against SARS-CoV-2 compared to healthy controls.

**Results:**

We show that a mean treatment duration of three months in the Ofa group led to near complete B cell depletion in line with altered composition of certain CD4^+^ T cell subpopulations such as enhanced frequencies of naive and a decrease of non-suppressive regulatory T cells (Tregs). Titer and neutralization capacity against SARS-CoV-2 variants was impaired while cellular immune response was sustained, characterized by a strong T helper 1 profile (Th1).

**Interpretation:**

In summary, low dosage ofatumumab treatment elicits sustained depletion of B cells in line with alterations of immune cells, mainly Tregs. This is associated with impaired humoral immune response towards SARS-CoV-2 vaccination but preserved, Th1 driven cellular immunity adding crucial information regarding early effects of low dosage anti-CD20 therapy on humoral and cellular immunity.

## Introduction

The novel virus SARS-CoV-2 can induce Coronavirus disease 2019 (COVID-19), a potentially life-threatening disease, which led to a global pandemic since early 2020. Soon, patients with compromised immunity either through a disorder or due to the need of immunosuppressive therapy got into focus regarding both their risk of infection and the risk of a fatal outcome as well as the immune response towards vaccination. Especially patients treated with anti-CD20 (aCD20) monoclonal antibodies such as rituximab or ocrelizumab, established as therapies in Multiple Sclerosis (MS) (1, 2), are at higher risk for hospitalization and a more severe course of COVID-19 (3). Moreover, we learned from a broad range of studies that the humoral immune response is worse in patients treated with aCD20 treatment (4). While the humoral immune response, prone to immune escape, is impaired, it could be established since fall 2021 that MS patients treated with aCD20 therapy elicit a robust T cell response, characterized mainly by CD8^+^ T cells (5).

A new treatment concept in MS recently having received market authorization is the application of ofatumumab, a fully human monoclonal anti-CD20-directed antibody applied in low dosages of 20 mg every four weeks subcutaneously. So far, it remains unclear how the initiation of a low dose aCD20 treatment regime might impact on the immune cell composition as well as humoral and cellular immune response towards SARS-CoV-2 vaccination. We therefore set out to analyze early ofatumumab treated patients regarding immunophenotyping as well as immune response following SARS-CoV-2 booster vaccination focusing on humoral immune response (neutralization capacity against wild type, Delta and the now dominating Omicron variant and titers) and deep immunophenotyping of T cell subsets. Those data will inform about vaccination strategies in low dosage aCD20 therapy.

## Methods

### Study design

The local ethics committee of the Ruhr-University Bochum authorized the study (20-6953-bio, 21-7351). We included n=9 healthy age-matched controls (HC) and n=10 ofatumumab treated MS patients (Ofa) recruited at the Department of Neurology, Ruhr-University Bochum, St. Josef-Hospital. Patients were boostered between September and December 2021. **Table 1** presents the associated demographics. All participants provided written informed consent. The samples were collected within 4-13 weeks following the third vaccination against SARS-CoV-2.

**Table 1:** Demographic and clinical characteristics of recruited MS patients and healthy controls. Age, disease duration (in years), Expanded Disability Status Scale (EDSS) and duration under therapy (in months) are presented as mean. SD: standard deviation, SEM: standard error of the mean. COVID: Coronavirus Disease 2019. DMF: dimethyl fumarate. Booster: third SARS-CoV-2 vaccination.

	MS	HC
Number	10	9
Male (%)	3 (30%)	2 (22%)
Female (%)	8 (70%)	7 (78%)
Age ± SD (range)	45 ± 11 (28 to 63)	41,5 ± 9,3 (26 to 50)
Disease duration ± SD (range)	12.6 ± 9.9 (3 to 33)	NA
Disease activity (during last 6 months)		
Relapse	2 (20%)	NA
No relapse	8 (80%)	NA
EDSS ± SD (range)	2,3 ± 1,2 (1 to 4)	NA
EDSS median	2	NA
EDSS ± SEM	2,3 ± 0,37	NA
COVID prior blood sampling	–	–
**Treatment**	**MS total 10**	**HC total 9**
**During first and second vaccination**		
DMF	2 (20%)	–
Interferon beta-1a	1 (10%)	–
Natalizumab	2 (20%)	–
Cladribrin	1 (10%)	–
Fingolimod	2 (20%)	–
Ozanimod	1 (10%)	–
No therapy	1 (10%)	9 (100%)
**During Booster**		
Ofatumumab	10 (100%)	–
Duration under therapy ± SD (range)	3,5 ± 0,7 (2 to 4)	–
no therapy	–	9 (100%)
**Vaccination**	**MS total 10**	**HC total 9**
**Basic Immunisation**		
Comirnaty (Pfizer/Biontech)	10 (100%)	6 (67%)
Vaxzevria (Astrazeneca)	–	–
Spikevax (Moderna)	–	2 (22%)
Mixed (Vaxzevria and Comirnaty)	–	1 (11%)
**Booster**		
Comirnaty (Pfizer/Biontech)	8 (80%)	7 (78%)
Spikevax (Moderna)	2 (20%)	2 (22%)

### Cell isolation and cryopreservation

Peripheral blood mononuclear cells (PBMCs) were isolated from the study participants’ blood withdrawn with 7.5 ml KABEVETTE^®^ G EDTA tubes after the booster vaccination. 30 mL blood-containing sample was layered on 15 ml ROTI^®^Sep 1077 human density gradient (Carl Roth) and centrifuged at 800 g for 30 min. without break. The PBMCs in the interface were washed twice and the pellet was resuspended in 10 mL PBS. Following cell counting, 10-20*10^6^ cells were immersed in 1 ml CTL-Cryo-ABC Freezing media Kit (ImmunoSpot^®^) for cryopreservation and stored at -80 °C.

### Immune cell phenotyping

For analysis of the immune cell composition without peptide stimulation, we used PBMCs of the same batches used for peptide stimulation for a detailed phenotyping of immune cell subpopulations *via* flow cytometry. Of the remaining cell suspensions 2.5*10^6^ cells were seeded in 2 mL OpTmizer™ CTS™ (Thermofisher) in a 12-well plate following resting for 1-2 h. For immunophenotype analysis, 2.5*10^6^ PBMCs were stained with viability stain LIVE/DEAD™ Fixable Blue Dead Cell Stain Kit for UV excitation (L23105, Thermo Fisher) for 30 min. at 4°C. Afterwards, Fc receptors were blocked by incubating the cells with Human TruStain FcX™ (422302, Biolegend) for 15 min. at 4°C. Subsequently, surface markers were stained for 15 min. at 4°C. Fixation and permeabilization were performed with Foxp3 staining buffer set (130-093-142, Miltenyi Biotec). Next, intracellular proteins Ki-67 and Foxp3 were stained for 30 min. at 4 °C. Antibody information is presented in **Table S1**. Flow cytometry measurement was performed using a Cytoflex LX (Beckman Coulter). Whole samples (2.5 *10^6^ PBMCs seeded) were recorded during flow cytometry measurement. The average of total events recorded was 1,422,939 (range [max – min]: 2,802,588 – 220,784) and the average of living cells recorded was 711,543 (range: 1,250,000 – 114.109) events. Regarding the analysis of B cells, an average of 63.262 (Range: 86237 - 28.771) events were recorded for the HC group and an average of 755 (Range: 3262 – 119) for the Ofa group. Flow cytometry data were analyzed with FlowJo™ (Becton Dickinson & Company, version 10.8.0). Barnes-Hut algorithm was implemented to generate t-Distributed Stochastic Neighbor Embedding (t-SNE) plots with FlowJo™ for high-dimensional data visualization. The t-SNE plots represent the distribution of the Treg cell subpopulations (nTreg, nsTreg, eTreg) and protein expression within the Treg cells of the respective groups. The Treg populations from all samples were used to create a single concatenated file. Pre-gating was done according to gating strategy (**Figure S1**): Time gate -> Single cells (FSC) -> Single cells (SSC) -> Viable cells -> CD3^+^ -> CD4^+^ -> Treg (CD127^low^ Foxp3^+^). Downsampling was performed to balance the two groups for fair comparison (59168 events per group). All markers, except non-relevant/excluded markers (Viability, CD8, CD19), were used for the calculation using Barnes-Hut algorithm in FlowJo. The gating strategy is presented in **Figure S1**.

### Anti-SARS-CoV-2 antibody titer

The enzyme-linked immunosorbent assay for determination of anti-SARS-CoV-2 spike antibody titers was conducted as described before (6). Briefly, the RBD sequence (spike glycoprotein amino acids 319–541) of the Wuhan strain of SARS-CoV-2 with a C-terminal HIS-tag was expressed in HEK 293-F cells and purified on a HisTrap Excel column using an ÄKTAxpress purification system. Ninety-six well flat bottom plates (maxisorp; Nunc) were coated with 3 μg/mL of SARS-CoV-2 spike RBD overnight at 4°C, following washing, blocking and incubation with diluted serum samples. The S Antibody (humanized anti-Spike antibody by Dianova/Cusabio, Stock concentration: 0.3 mg/mL) was used as positive control (1:5,000 final dilution) and calibrator (1:40,000 final dilution). HRP conjugated anti-human IgG (Dianova) was used as secondary antibody. Antibody titers were calculated as BAU/ml and values higher than 30.3 were considered as positive (6).

Anti-SARS-CoV-2 nucleocapsid (NCP) antibody titer was determined using the Anti-SARS-CoV-2-NCP ELISA (IgG) (EUROIMMUN) following the manufacturer’s instructions to exclude unremarked prior infections which could have affected the immune response. A ratio of the OD of the sample over the OD of the included calibrator of <0.8 is considered negative, ≥ 0.8 to <1.1 borderline and a ratio ≥1.1 positive.

### SARS-CoV-2 neutralization assay

We used previously described pseudoviruses (7). Complement factors in sera were inactivated (30 min. at 56 °C). The sera were incubated as quadruplicates of twofold serial dilutions (1:20 to 1:2560) in 96 well plates with single cycle VSV∗ΔG(FLuc) pseudoviruses bearing the SARS-CoV-2 spike WT (D614G) protein (8), SARS-CoV-2 B.1.617.2 (Delta) (EPI_ISL_1921353) spike protein or SARS-CoV-2 B.1.1.529 (Omicron) (EPI_ISL_6640919) spike in the envelope prior to infection of Vero E6 cells (1x10^4^ cells/well) in DMEM with 10% FBS (Life Technologies) for 18 h. Subsequently, the reporter activity of the firefly luciferase (FLuc) was determined with a CentroXS LB960 (Berthold) as described previously (9). The pseudovirus neutralization dose 50% (PVND50) was calculated as the reciprocal antibody dilution causing 50% inhibition of the luciferase reporter with a detection range between 1:20 to 1:2560.

### PBMC stimulation using SARS-CoV-2 peptide pool

For PBMC restimulation with SARS-CoV-2 peptide mix, the cryopreserved cells were thawed, counted and 80 µL of a 2.5*10^6^ cells per ml cell suspension seeded in duplicates in a 96 well plate in OpTmizer ™ CTS ™ (Thermofisher) medium (10). Following a resting time of 2 hours (h), the stimulation was induced with 2 µg/ml PepMix SARS-CoV-2 (JPT) for 16-18 h under the presence of Brefeldin A solution (BioLegend). The 315 (158 + 157) peptides in the peptide mix were derived of the spike glycoprotein of SARS-CoV-2 (Swiss-Prot ID: P0DTC2). Furthermore, unstimulated controls of all samples in duplicates were seeded and incubated without PepMix SARS-CoV-2. For positive controls, three samples each of HC and Ofa were seeded and stimulated with 4 µL CytoStim™ (Miltenyi Biotec, 130-128-034).

### Flow cytometry of stimulated PBMCs

The PepMix SARS-CoV-2-stimulated cells were stained for flow cytometry starting with a live dead stain for 10 min. with blue fluorescent reactive dye for UV excitation (Invitrogen). Subsequently, cells were labelled against extracellular markers (BioLegend: anti-CD3 Cat. No. 300448, anti-CD4 Cat-No. 344608, anti-CD8a Cat. No. 344746, anti-CD19 Cat. No. 302226, anti-CD137 Cat. No. 309826, anti-CD154 Cat. No. 310832, anti-CD14 Cat. No. 367144; for dilutions see **Table S1**) for 30 min. at 4°C and intracellular markers (BioLegend, anti-IFN-γ Cat. No. 502509, anti-IL-2 Cat. No. 500310, anti-IL-4 Cat. No. 500828, anti-IL-17A Cat. No. 512338; for dilutions see **Table S1**) for 30 min. at room temperature. Prior to intracellular staining, the cells were fixed and permeabilized with Fixation Buffer (BioLegend) and intracellular staining perm wash buffer (BioLegend). Flow cytometry was performed using a Cytoflex LX (Beckman Coulter). The gating strategy is shown in **Figure S2**. The threshold levels of the gates for the cytokines were set using unstimulated controls and the CytoStim™ stimulated positive controls. Flow cytometry data were analyzed with FlowJo™ (BD, version 10.7.1).

### Statistics

Statistical analysis was conducted as indicated in respective figure legends using GraphPad Prism (version 9.2.0). Since most of the data did not follow a normal distribution the data of the two groups were analyzed using two-tailed Mann-Whitney U test. Correlations were analyzed using Spearman correlation. P<0.05 was considered as statistically significant. Significances were presented as *p<0.05; **p<0.01; ***p<0.001 and ****p < 0.0001.

### Data Availability

All data are available from the corresponding author SF upon reasonable request.

## Results

### Baseline characteristics

**Figure 1:**
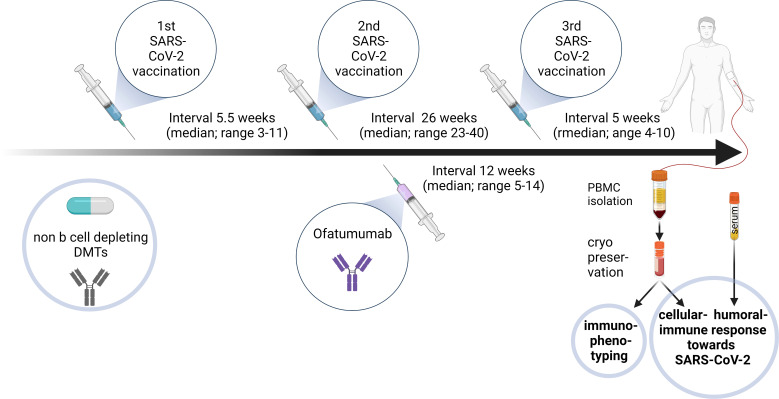
Diagram outlining the study design for ofatumumab treated MS patients. 9/10 relapsing remitting multiple sclerosis (RRMS) patients were under different disease modifying therapies (DMTs) during the first and second SARS-CoV-2 vaccinations, conducted in a median 5.5-week interval. After the second vaccination ofatumumab was initiated before the third vaccination (median 26 weeks after second vaccination and median 12 weeks after first ofatumumab administration). Blood was sampled 5 weeks (median) after the booster vaccination. Peripheral blood mononuclear cells (PBMCs) and serum were isolated and cryopreserved. PBMCs were used for immunophenotyping. Humoral and cellular immune response towards SARS-CoV-2 restimulation were assessed in different assays. Created with BioRender.com.

Ofatumumab treated MS patients (Ofa) were 45 ± 11.8 (28 to 63) years old compared to 41.1 ± 9.9 years (26 to 55) in healthy controls (HC). MS disease duration was 13.9 ± 10.5 (3 to 33) years and patients were under treatment with ofatumumab for 3.5 ± 0.7 (2 to 4) months. All patients of both groups had been boostered with mRNA vaccination. During basic immunization (first and second vaccination) with Comirnaty (100%) MS patients were on non B cell depleting disease modifying therapies as shown in **Table 1**. Three of those patients had been treated with S1P receptor modulators. Ofatumumab treatment started between the second and third vaccination. Patients were contacted 7-9 months following third immunization and 4/10 patients reported that they had a breakthrough infection. The severity of COVID was mild to moderate (4/4 WHO score 2) and the most common symptoms were sore throat, cough and fever. An overview of the study design is given in **Figure 1**.

### Altered lymphocyte composition in ofatumumab treated MS patients

**Figure 2:**
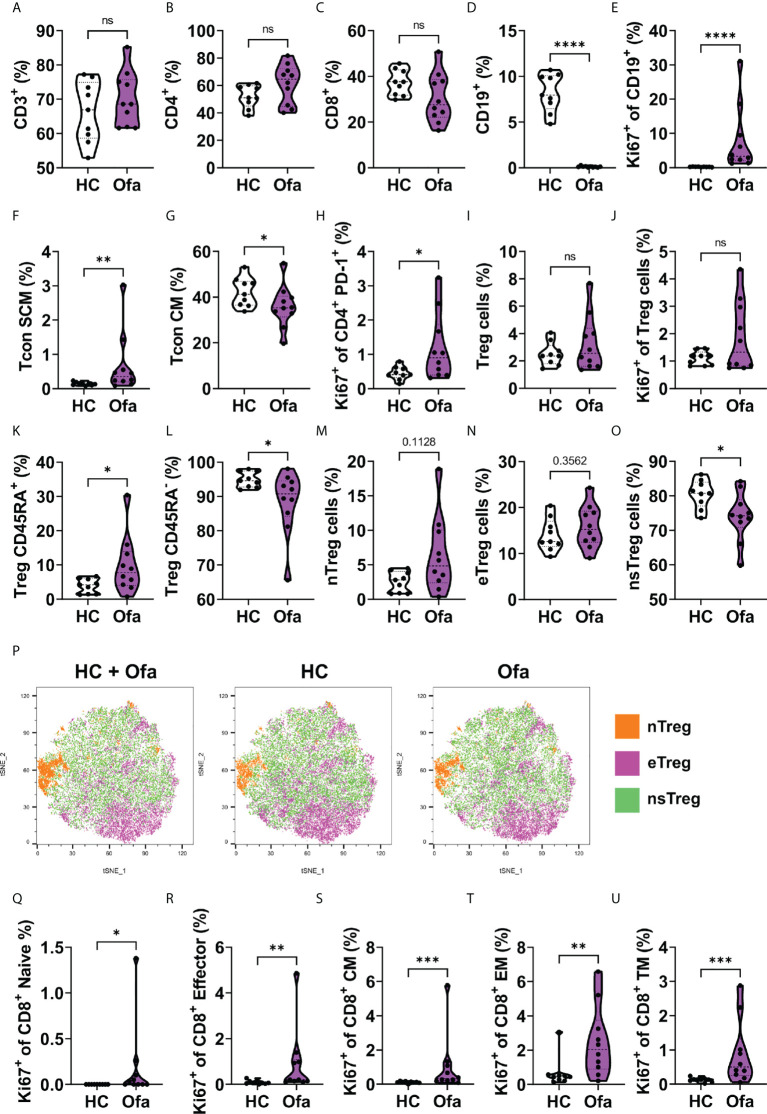
Lymphocyte composition in ofatumumab treated MS patients and healthy controls. Immunophenotyping was conducted in Ofa and HC using multi-color FACS. **(A-D)** T cell frequencies showed no difference between Ofa and HC but a tendency towards higher frequencies of T helper cells and less cytotoxic T cells in the Ofa group. **(D, E)** Ofa patients had significantly lower frequencies of B cells with near complete depletion (p<0.0001) compared to HC with higher proliferation of remaining B cells as shown with higher expression of Ki67 (p<0.0001). **(F-O)** T helper cell subpopulation analyses with specific focus on regulatory T cells (Tregs) including unsupervised clustering of the Treg populations **(P)**. **(Q-U)** proliferation of cytotoxic T cell subpopulations was significantly higher in the Ofa group. Tcon SCM: stem cell like memory T conventional cells. Tcon CM: central memory T conventional cells. Treg: T regulatory. nTreg cells: naïve regulatory T cells. eTreg cells: effector regulatory T cells. nsTreg cells: non-supressive regulatory T cells. TM: transitional memory cells. Data were analyzed with a two-tailed Mann-Whitney U test. n=10 Ofa, n=9 HC. *p<0.05; **p<0.01; ***p<0.001; ****p < 0.0001, ns, not significant.

We examined blood immune cell compositions to determine changes induced by ofatumumab treatment. B and T cell composition in Ofa and HC were analyzed using multi-parametric flow cytometry. No differences in CD3^+^ T cell frequencies were found between the two groups (**Figure 2A**). Likewise, we did not detect major differences in the frequencies of CD4^+^ T helper and CD8^+^ cytotoxic T cells, although there was a slight increase in CD4^+^ T cell frequencies and a corresponding slight decrease in CD8^+^ T cell frequencies in the Ofa group (**Figures 2B, C**). Unsurprisingly, B cells were barely detectable in the Ofa group (**Figure 2D**), while the remaining B cells displayed enhanced proliferation as analyzed by the expression of Ki-67 (**Figure 2E**), presumably attempting the recolonization of the empty B cell niche. Next, we examined the composition of the CD4^+^ conventional T (Tcon) cell subpopulation. Both groups exhibited equal frequencies of naïve Tcon cells (**Figure S3B**). Similarly, the frequencies of CD25^+^ Tcon cells remained equal between the two groups, despite the elevated percentage of Ki-67-expressing naïve Tcon cells found in treated MS patients (**Figures S3A, C**). Contrariwise, stem cell-like memory Tcon cell frequencies were higher in the Ofa group (**Figure 2F**), although the proliferation rate was similar to the control group (**Figure S3D**). We also found reduced frequencies of central memory Tcon cells in the group of treated MS patients (**Figure 2G**) despite their slightly elevated proliferation rate (**Figure S3E**). Although we detected a prominent proliferation in transitional memory, effector memory and terminally differentiated Tcon cells in the Ofa group, this was not reflected in an increase in the percentages of the respective cell populations (**Figures S3F–K**). We found a similar phenomenon when we analyzed PD-1 expressing CD4^+^ T cells that exhibited a high proliferation rate (**Figure 2H**), but identical frequencies (**Figure S3L**) in treated MS patients. Strikingly, despite the elevated percentage of Ki-67 expressing circulating T follicular helper cells (cTFH), the frequency of this population was diminished in the Ofa group (**Figure S3M**). Circulating T follicular regulatory cells (cTFR) showed a tendency towards being upregulated in the Ofa group and the ratio of cTFH and cTFR was significantly lower in the Ofa group (**Figures S3O, P**). Next, we analyzed the CD127^low^ Foxp3^+^ regulatory T (Treg) cell compartment. Again, while the percentages of Treg cells were similar between the groups (**Figure 2I**), the Treg cells of the Ofa group presented a tendency of elevated proliferation (**Figure 2J**). Interestingly, the percentage of CD45RA^+^ Treg cells (naïve/resting Treg) and CD45RA^-^ Treg cells (activated/effector Treg) in the Ofa group was higher and lower, respectively, compared to HC (**Figures 2K, L**). When we examined the Treg cell subpopulations in more detail, we observed elevated frequencies of naïve Treg cells (**Figure 2M**) with no effect on effector Treg cells in the Ofa group (**Figure 2N**). In contrast, this group had a lower percentage of non-suppressive Treg cells compared to HC (**Figure 2O**). These observations were supported by unsupervised clustering using t-SNE plots to visualize the distribution of Treg cell subpopulations (**Figure 2P**; **Figure S4**).

Subsequently, we compared the composition of the CD8^+^ cytotoxic T cell lineage between the two groups. Neither the PD-1 expressing nor the CD107a (LAMP-1) expressing CD8^+^ T cell frequencies presented differences (**Figures S5A, B**). In the same line, we could not observe differences in the frequencies of the CD8^+^ T cell subpopulations, i.e. naïve, stem cell-like memory, central memory, transitional memory, effector memory and effector CD8^+^ T cells (**Figures S5C–H**). Of note, regardless of the equal composition of the different CD8^+^ T cell subsets, we detected that several of these subpopulations exhibited elevated proliferation rates in the Ofa group (**Figures 2Q–U** and **Figure S5I**).

Taken together, besides the expectable B cell depletion caused by the treatment, we found that the composition of certain CD4^+^ T cells subpopulations was altered in ofatumumab treated MS patients. Furthermore, a considerable number of T cell subsets presented features of enhanced proliferation in these patients.

### Blood serum’s neutralization capacity is dependent on anti-SARS-CoV-2-spike antibody titer

**Figure 3:**
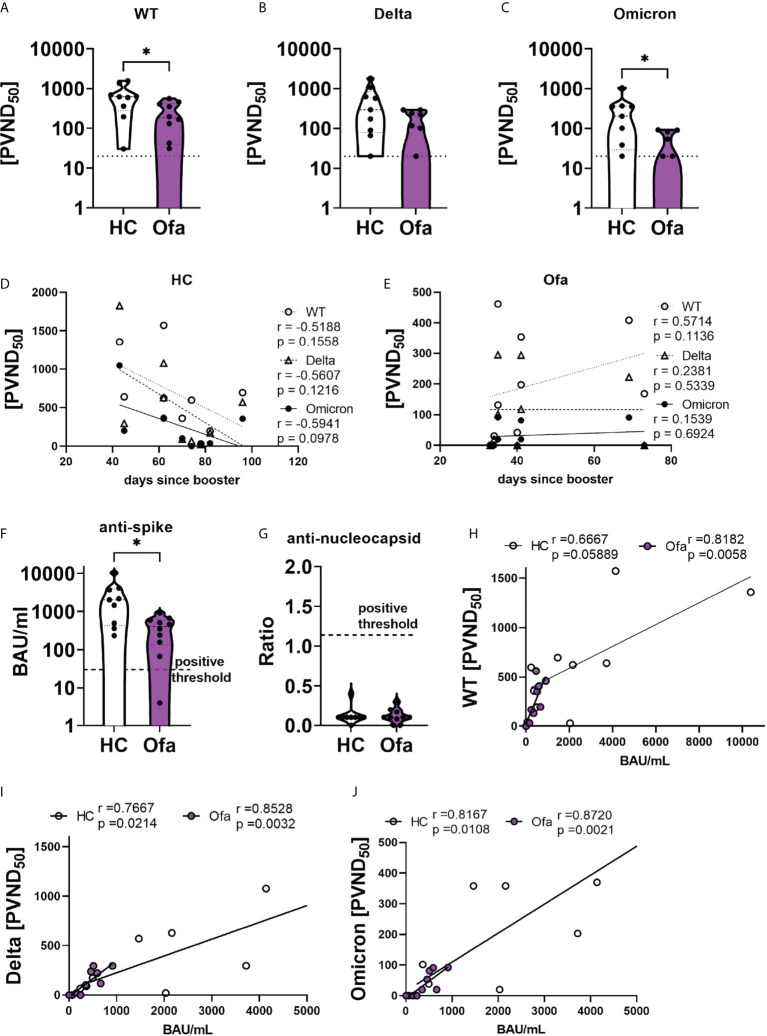
Ofatumumab treated MS patients elicit lower SARS-CoV-2 spike titers and less neutralizing capacity. Sera of Ofatumumab treated MS patients (Ofa) and healthy controls (HC) were collected 4-13 weeks following the third vaccination against SARS-CoV-2 and neutralization against SARS-CoV-2 variants and anti-SARS-CoV-2-spike titers were measured. **(A–C)** Sera of Ofa treated MS patients have significantly lower neutralization capacity against a) wild type (WT, p=0.0220) and **(C)** Omicron (p=0.0208) variants, with borderline significance against the **(B)** Delta variant (p=0.0677) of the SARS-CoV-2 virus compared to HC. **(D)** Neutralizing capacities of HCs did not depend on the number of days since the third vaccination. **(E)** In the Ofa group, the neutralization of the WT variant was higher the more days had elapsed since the third vaccination. The neutralization of the Delta and Omicron variant did not depend on the days since booster. **(F)** anti-SARS-CoV-2-spike titers were lower in Ofa patients (p=0.0172) compared to HC. **(G)** No patient had anti-SARS-CoV-2 nucleocapsid antibodies. The anti-SARS-CoV-2-spike titers strongly correlated with neutralization capacity against **(H)** WT, **(I)** Delta and **(J)** Omicron. BAU/mL: binding antibody unit per milliliter. PVND_50_: pseudovirus neutralization dose 50%. Data were analyzed with a two-tailed Mann-Whitney U test and Spearman correlation. n=10 Ofa, n=9 HC. *p<0.05.

We then analyzed the humoral immune response following SARS-CoV-2 vaccination. First we confirmed that none of the study participants had a previous infection with SARS-CoV-2 by SARS-CoV-2 N ELISA (**Figure 3G**). Using a cell-based assay we depicted a reduced neutralization capacity in the Ofa group compared to HC against wild type (WT, p=0.0220), Delta with borderline significance (p=0.0677) and Omicron (p=0.0208) variants (**Figures 3A–C**). In line with those findings, anti-SARS-CoV-2 spike titers of Ofa treated MS patients were lower compared to HC (p=0.0172) (**Figure 3F**). Of note, the antibody levels of the Ofa group were apart from one sample above the positive threshold of 30.3 BAU/mL. We also collected data prior to and after the booster generated with the Elecsys^®^ immunoassay (Roche), which were, however, not available for every patient at two time points. Prior to booster 5/10 MS patients had a mean titer of 498.6 U/mL (max 2500 U/ml), while 5/10 MS patients had a mean titer of 1100.7 U/mL after booster. There were only data of two longitudinal patients for both time points, showing an increase by 237.3% and 643.3% following booster vaccination. Of note, anti-nucleocapsid antibodies were negative in both groups, suggesting that there was no inapparent COVID-19 disease which could have influenced the immune response (**Figure 3G**). To understand dynamics of humoral response over time we correlated the neutralization capacity with time since booster vaccination. In HC, the neutralization capacity did not correlate with the days elapsed between the booster vaccination and blood sampling. Despite the absent significant correlation, there was a trend for decreasing neutralization capacity with increasing time since the booster in the HC group (r=-0.52 - -0.59; **Figure 3D**). In the Ofa group there was also no correlation for Delta and Omicron neutralization. Surprisingly, neutralization against WT was stronger with rising interval following booster (r=0.56; p=0.1136; **Figure 3E**). As expected, the neutralizing capacity strongly depended on the anti-SARS-CoV-2 spike titer both in Ofa treated MS patients and HC for WT, Delta and Omicron (**Figures 3H-J**). The frequency of B cells did not correlate with the SARS-CoV-2 spike titer and the neutralization capacity as well as the cellular immune response against SARS-CoV-2 peptide mix stimulation (**Figures S7A, B, E, F**). We could detect a trend towards correlation of the frequency of Tregs determined by immunophenotyping with the humoral immune response (**Figures S7C, D**) of which only the neutralizing capacity against Omicron variant correlated significantly with Treg frequencies.

### Lower frequency of cytotoxic T cells in ofatumumab treated MS patients

**Figure 4:**
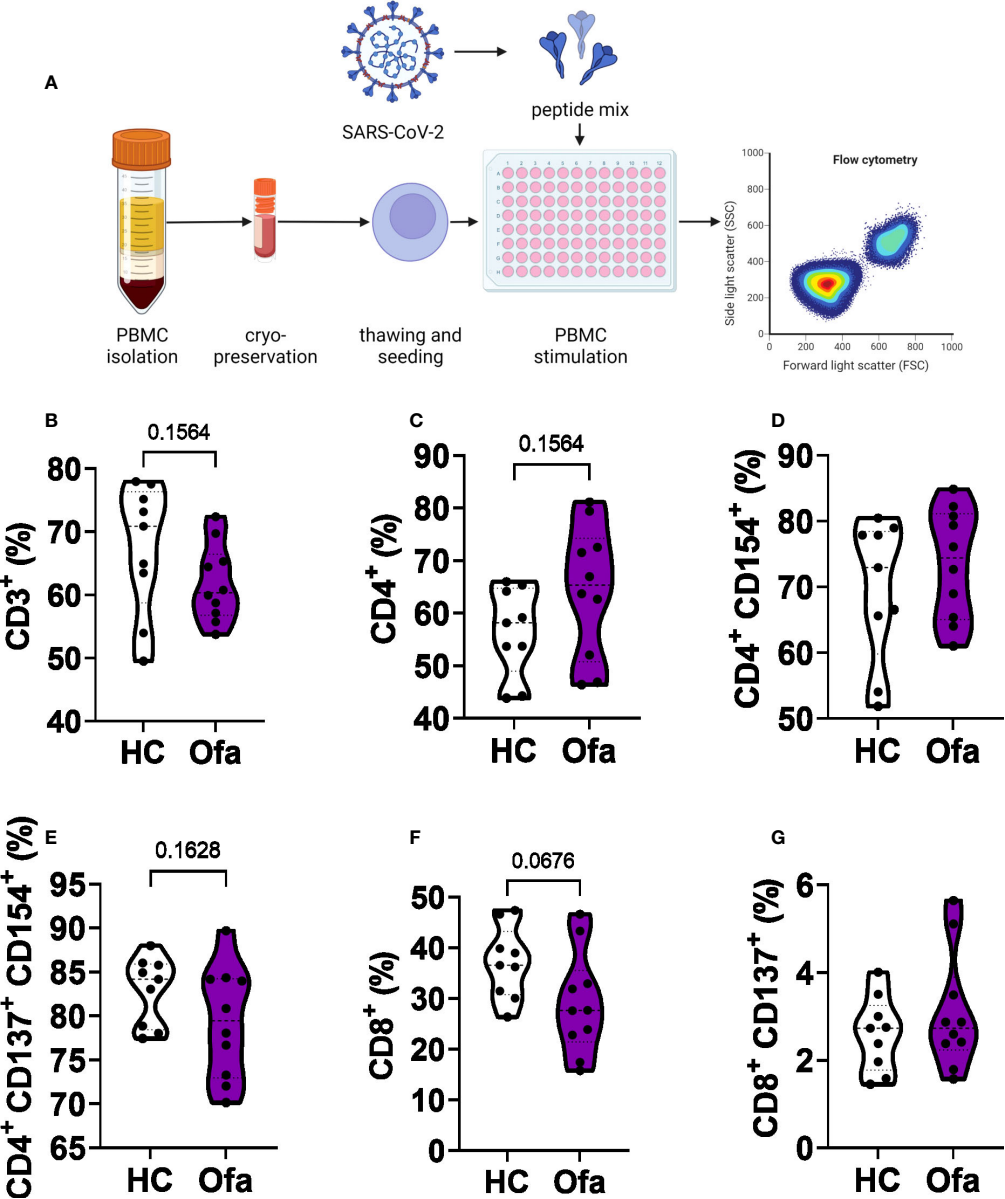
Flow cytometry analysis of T cell populations. **(A)** peripheral blood mononuclear cells (PBMCs) of Ofatumumab treated MS patients (Ofa) and healthy controls **(HC)** were collected 4-13 weeks following the third vaccination against SARS-CoV-2 and restimulated with a SARS-CoV-2 peptide pool followed by immunophenotyping *via* flow cytometry. SARS-CoV-2 peptide stimulated PBMCs showed **(B)** no altered frequencies of CD3^+^ T cells (p=0.1564) and **(C)** CD4^+^ T helper cells or **(D, E)** activated CD4^+^ T helper cells. **(F)** Frequencies of CD8^+^ T cells were lower in the Ofa group compared to HC (p=0.0676) while **(G)** activated Tc cells did not differ. Data were analyzed with a two-tailed Mann-Whitney U test. n=10 Ofa, n=9 HC. **(A)** Created with BioRender.com.

Following the identification of suspected impaired humoral response in Ofa treated MS patients as well as differences of immune cell populations between the two groups, we set out to analyze T cell responses following stimulation with a SARS-CoV-2 peptide mix (**Figure 4A**). After stimulation, we analyzed different cell populations *via* flow cytometry. While overall frequencies of CD3^+^ T cells were unaffected (**Figure 4B**) we found a trend towards higher frequencies of CD4^+^ T cells (Th) in Ofa treated patients (**Figure 4C**, p=0.1564) in accordance with a trend towards lower frequencies of CD8^+^ cytotoxic T cells (Tc; p=0.0676; **Figure 4F**). Frequencies of activated Th or Tc cells did not differ while overall frequencies of activated Tc cells were below 10% (**Figure 4G**) in both groups compared to more than 50% activated Th cells (**Figures 4D, E**).

### Th1 cell populations were significantly increased in MS patients under ofatumumab therapy

**Figure 5:**
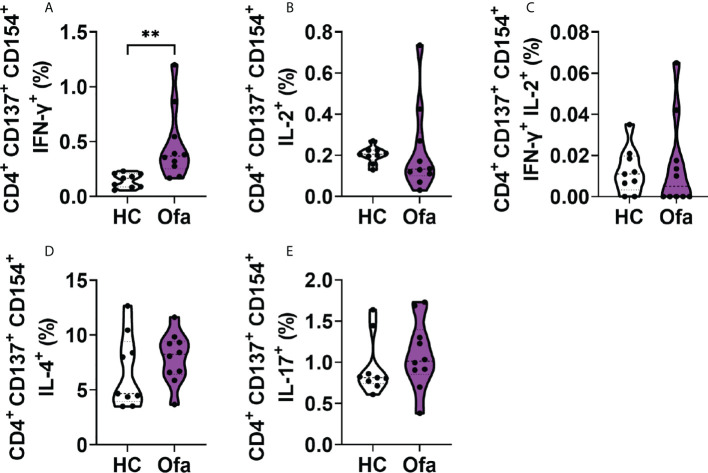
Ofa treated patients exhibit a stronger response of IFN-γ^+^ activated T helper cells. Peripheral blood mononuclear cells (PBMCs) of ofatumumab treated MS patients (Ofa) and healthy controls **(HC)** were collected 4-13 weeks following the third vaccination against SARS-CoV-2 and restimulated with a SARS-CoV-2 peptide pool following immunophenotyping *via* FACS. **(A)** Frequencies of CD4^+^ IFN-γ^+^ T cells were significantly higher in the Ofa group compared to HC (p<0.01). **(B)** The expression of IL-2 (p=0.2343), **(C)** IFN-γ and IL-2 (p=0.6513), **(D)** IL-4 (p=0.2775) and **(E)** IL-17 (p=0.2110) in CD4^+^ T helper cells did not differ between respective groups. Data were analyzed with a two-tailed Mann-Whitney U test. n=10 Ofa, n=9 HC. **p < 0.01.

To analyze the responses of T cells towards SARS-CoV-2 activation, we analyzed cytokine expressions in activated Th cells. For this analysis with focused on activated Th cells expressing both CD154, the general CD4^+^ specific activation marker together with CD137. Amongst activated Th cells, IFN-γ was significantly more expressed in Ofa treated MS patients (**Figure 5A**). Activated Th cells expressing IL-2 alone as well as IFN-γ/IL-2 double positive cells were not differently expressed between both groups (**Figures 5B, C**). This was also the case for IL-4 and IL-17 expressing Th cells, which did not differ (**Figures 5D, E**). CD154^+^ Th cells without expression of CD137 showed the same effects (**Figure S6**).

### Cytotoxic T cells expressing IFN-γ or both IFN-γ and IL-2 showed lower frequencies in ofatumumab treated MS patients

**Figure 6:**
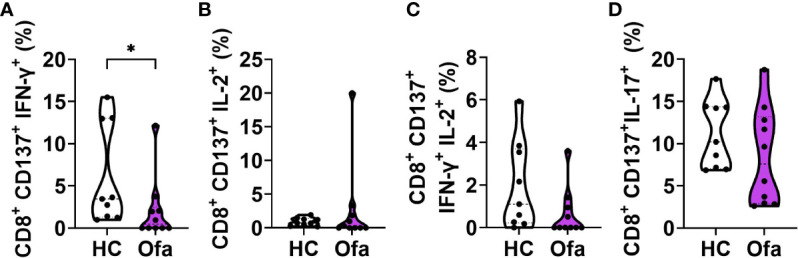
Ofa treated MS patients express lower response of IFN-γ^+^ and IFN-γ^+^IL-2^+^ in cytotoxic T cells. Peripheral blood mononuclear cells (PBMCs) of ofatumumab treated MS patients (Ofa) and healthy controls **(HC)** were collected 4-13 weeks following third vaccination against SARS-CoV-2 and restimulated with a SARS-CoV-2 peptide pool following immunophenotyping *via* FACS. **(A)** Frequencies of IFN-γ^+^ cytotoxic T cells and **(C)** IFN-γ/IL-2 double positive cytotoxic T cells were significantly lower in the Ofa group compared to HC. Expression of **(B)** IL-2^+^ cells and **(D)** IL-17^+^ cytotoxic T cells did not differ. Data were analyzed with a two-tailed Mann-Whitney U test. n=10 Ofa, n=9 HC. *p<0.05.

Since overall frequencies of CD8^+^ T cells were reduced in Ofa treated MS patients we set out to investigate subpopulations of activated cytotoxic T cells (Tc). In line with reduced overall frequencies we found reduced frequencies of IFN-γ expressing Tc cells in Ofa treated MS patients (p<0.05; **Figure 6A**), while IL-2, IFN-g/IL-2 and IL-17 expressing Tc cells did not differ (**Figures 6B–D**). In the HC group, the frequencies of IL-17 expressing CD4^+^ and CD8^+^ T cells after the peptide mix stimulation were dependent on the percentage of Tregs present before peptide stimulation (**Figure S7G**). In the Ofa group, CD4^+^ IFN- γ^+^ cells correlated with Treg frequencies (**Figure S7H**).

## Discussion

Since it is known that Treg cells are able to limit inflammatory processes in MS (11), we assessed the distribution of different Treg subpopulations after of atumumab treatment. Interestingly, there was an increase of CD45RA-expressing regulatory T cells as well as a reduction of non-suppressive T reg cells in Ofa treated MS patients, suggesting effects on T cell regulation as additional mediator of ofatumumab mechanism of action besides B cell depletion. The Treg cell population was gated on CD127^low^ Foxp3^+^ cells as these expression profiles are associated with autoimmunity in MS. These cells include nsTregs, considered as inflammatory due to secretion of inflammatory cytokines, described in MS (12). The reduction of Treg cells in the Ofa group could thus potentially be an anti-inflammatory effect in case inflammatory T reg cells would be reduced, hence decreasing the whole Treg population; an aspect which should be investigated further.

Immunosuppression is a massive challenge for the management of patients with chronic conditions such as MS with aCD20-directed therapies, having induced severe concerns regarding immune response towards SARS-CoV-2 vaccination. Humoral and cellular immune response in early treated patients is so far incompletely understood. Moreover, it remains unknown how early ofatumumab treatment might impact on immune cell subsets. We therefore assessed the lymphocyte composition in Ofa treated MS patients. We found a strong upregulation of proliferation markers in B cells, suggesting a compensatory mechanism following depletion to fill the B cell niche.

We then assessed the immune response towards SARS-CoV-2. We show that, in line with previous reports from patients treated with other aCD20-directed monoclonal antibodies such as ocrelizumab and rituximab, ofatumumab treated patients elicit an impaired humoral immune response both regarding spike-Abs and neutralization *in vitro*, although patients were under aCD20 therapy for only 2-4 months. All patients generated a CD4^+^ and CD8^+^ T cell response with higher expression of Th1 cells compared to controls. Contrarily, it has been shown that patients under aCD20 treatment applied in 6-month infusion cycles with mean 3.2 prior infusion cycles elicit a skewed T cell response comprising of circulating follicular helper cells and increased CD8^+^ T cell response (5), suggesting that this might be a compensatory mechanism following long-term treatment, not yet observed in initially treated Ofa patients. We assume that both the short term B cell depletion only conducted over some months in low dosages and maybe effects of ofatumumab itself might have shaped this specific response.

So far, there is limited evidence regarding the immune response following ofatumumab treated patients infected with SARS-CoV-2. The report of a patient followed in the ASCLEPIOS-extension study who was 42 months under ofatumumab showed effective IgM and IgG response against the spike protein following COVID-19 infection despite complete B cell depletion (13). Another case series of four patients documented strong T cell response in patients without titer against the spike protein following COVID-19 infection (14). Data derived from the phase 3 study ALITHIOS which investigated ofatumumab vs. teriflunomide in MS documented that 245 of 1703 patients (14.3%) reported a COVID-19 disease of whom 44.1% had a mild and 46.5% a moderate course (15). Only 9% had a severe or life-threatening disease; two patients died. Only in 15.9% ofatumumab was temporarily interrupted. Our data provide evidence, that already in early ofatumumab treated patients the humoral immune response is substantially impaired while cellular protection is still robust, presumably mainly *via* Th1 response. Strong Tc1 response documented in other studies (5) might be a result of compensatory higher proliferation of this compartment as shown in our study with high Ki67 expression in several lymphocyte subsets.

Limitations of the study presented here include the relatively small sample size, mostly caused by the limited number of patients boostered within the first months of ofatumumab treatment. Moreover, there were no samples available prior initiation of ofatumumab therapy which could have informed about the immune cell composition prior ofatumumab initiation as well as baseline data on humoral and cellular immunity. Most patients had been treated with prior DMTs, which might have influenced the immune cell composition. Moreover, there should have been a preformed memory formation of both B and T cells during first and second vaccination, presumably having been modulated by initial DMTs, forming the basis for the immune response following booster vaccination. Since booster vaccination leads to an enhanced humoral immune response even in B cell depleted patients, while complete B cell depletion is associated with decreased probability of seroconversion (OR 0.14; p = 0.021) (16), it might be possible that the humoral immune response measured here under ofatumumab treatment might have been due to third vaccination and not the levels achieved following second vaccination. This, however, is not supported by the data since robust longitudinal data are missing. However, none of the patients had been under B cell depletion prior ofatumumab treatment. We therefore believe that at least the B cell proliferation assessed using Ki-67 expression might mostly be due to the ofatumumab effect. Another limitation is related to the wide timespan of blood sampling following booster vaccination (10 MS patients recruited within 4-10 weeks; median 5 weeks), since some patients were hard to follow. Further limitations are related to the real-world situation with different intervals of the booster vaccination. Since the number of patients who had not only been vaccinated but who also had an infection is rising, immune response in patients with hybrid immunity (convalescent and vaccinated) will be the main group of patients to focus on in future studies.

So far, we provide, to the best of our knowledge, the first data set of SARS-CoV-2 immunization in ofatumumab treated MS patients. A phase 4 trial which will provide more data is currently ongoing (COMB157GDE01).

*In summary*, even short term ofatumumab treatment elicits an impaired humoral immune response in MS patients with preserved cellular immunity, characterized by a strong Th1 phenotype. Moreover, ofatumumab elicits an altered distribution of Treg subpopulations adding further evidence regarding effects on peripheral immunity using low dosage aCD20 directed treatment regimes.

## Data availability statement

The raw data supporting the conclusions of this article will be made available by the authors, without undue reservation.

## Ethics statement

The studies involving human participants were reviewed and approved by the local ethics committee of the Ruhr-University Bochum (20-6953-bio, 21-7351). The patients/participants provided their written informed consent to participate in this study.

## Author contributions

SF, PT, UC and KH acquired patients’ samples. NH, CP-S, PT, CB and DU performed the experiments. SF, NH, CP-S, DU and SP analyzed data. SF, NH and CP-S made the figures. SF, NH and CP-S wrote the first draft of the manuscript. CP-S, PT, UC, JM, CB, DU, CW, OO, AR-S, KH, SP, IS and RG corrected the manuscript. SF, OO, AR-S, KH provided ethics approval. SF and RG designed and supervised the study. RG acquired funding. All authors read and approved the final manuscript. All authors contributed to the article and approved the submitted version.

## Funding

This research was supported by a grant from the Rose foundation T298/28150/2016.

## Acknowledgments

We thank Gert Zimmer, Institute for Virology und Immunology, Switzerland and Department of Infectious Diseases and Pathobiology (DIP), Vetsuisse Faculty, University of Bern, Switzerland and Stefan Pöhlmann and Markus Hoffmann, Infection Biology Unit, German Primate Center - Leibniz Institute for Primate Research, Göttingen, Germany, Faculty of Biology and Psychology, Georg-August-University Göttingen, Göttingen, Germany, for providing SARS-CoV-2 spike plasmids and reagents. We acknowledge support by the Open Access Publication Funds of the Ruhr-University Bochum.

## Conflict of interest

SF has received speaker’s and/or scientific board honoraria from Biogen, BMS, Celgene, Genesis Pharma, Novartis and Roche and grant support from Ruhr-University Bochum, DMSG, Stiftung für therapeutische Forschung, Lead Discovery Center GmbH and Novartis. KH has received travel grants from Biogen, Novartis and Merck and received speaker and research honoraria from Biogen Idec Germany, Teva, Sanofi Genzyme, Novartis, Bayer Health-Care, Merck Serono and Roche. RG serves on scientific advisory boards for Teva Pharmaceutical Industries Ltd., Biogen, Bayer Schering Pharma, and Novartis; has received speaker honoraria from Biogen, Teva Pharmaceutical Industries Ltd., Bayer Schering Pharma, and Novartis; serves as editor for Therapeutic Advances in Neurological Diseases and on the editorial boards of Experimental Neurology and the Journal of Neuroimmunology; and receives research support from Teva Pharmaceutical Industries Ltd., Biogen Idec, Bayer Schering Pharma, Genzyme, Merck Serono, and Novartis.

The remaining authors declare that the research was conducted in the absence of any commercial or financial relationships that could be construed as a potential conflict of interest.

## Publisher’s note

All claims expressed in this article are solely those of the authors and do not necessarily represent those of their affiliated organizations, or those of the publisher, the editors and the reviewers. Any product that may be evaluated in this article, or claim that may be made by its manufacturer, is not guaranteed or endorsed by the publisher.

## Abbreviations

aCD20, anti-CD20; COVID-19, Coronavirus disease 2019; CM, Central memory; cTFH, Circulating T follicular helper; cTFR, circulating T follicular regulatory cells; EM, Effector-memory; eTreg, effector Treg; MS, Multiple sclerosis; N, Naïve; nTreg, Naïve Treg cells; nsTreg, Non-suppressive Treg; PBMCs, peripheral blood mononuclear cells; PVND50, Pseudovirus Neutralizing titer 50; SARS-CoV-2, Severe acute respiratory syndrome coronavirus type 2; SD, Standard deviation; SEM, Standard error of the mean; TM, Transitional memory; SCM, Stem cell-like memory; TD, Terminally differentiated.
