# Supplementary material for: Immune response in ofatumumab treated multiple sclerosis patients after SARS-CoV-2 vaccination
Source: Front Immunol. 2022 Aug 31;13:980526. doi: 10.3389/fimmu.2022.980526 (PMC9471319; doi:10.3389/fimmu.2022.980526)
Supplement: Supplementary file 1 [file DataSheet_1.pdf]

SUPPLEMENT

Immune Response in Ofatumumab treated Multiple Sclerosis Patients after SARS-CoV-2 Vaccination

Simon Faissner[1], Neele Heitmann[1], Carlos Plaza-Sirvent[2], Paulina Trendelenburg[1], Ulas Ceylan[1], Jeremias Motte[1], Clara Bessen[2], Doris Urlaub[3], Carsten Watzl[3], Oliver Overheu[4], Anke Reinacher-Schick[4], Kerstin Hellwig[1], Stephanie Pfaender[5], Ingo Schmitz[2], Ralf Gold[1]

[1] Department of Neurology, Ruhr-University Bochum, St. Josef-Hospital, Bochum, Germany,

[2] Department of Molecular Immunology, Ruhr-University Bochum, Bochum, Germany

[3] Department for Immunology, Leibniz Research Centre for Working Environment and Human Factors (IfADo) at TU Dortmund, Dortmund, Germany

[4] Department of Hematology, Oncology and Palliative Care, St. Josef Hospital, Ruhr-University Bochum, Bochum, Germany

[5] Department of Molecular and Medical Virology, Ruhr-University Bochum, Bochum, Germany

Supplementary Table 1: Antibodies used for immunostaining prior to flow cytometry. The top table presents the antibodies used for lymphocyte phenotyping. The table at the bottom includes the antibodies used after peripheral blood mononuclear cells restimulation.

antibody	conjugate/dye	dilution	clone	RRID
viability	blue fluorescent reactive dye	1/1000		
anti-CD3	BV510	1/100	UCHT1	AB_2563468
anti-CD4	PerCP-Cy5.5	1/500	OKT4	AB_1186124
anti-CD8a	APC/Fire750	1/500	RPA-T8	AB_2572095
anti-CD19	Alexa Fluor 700	1/500	HIB19	AB_493751
anti-CD25	BV421	1/200	BC96	AB_10896914
anti-CD27	BV785	1/100	O323	AB_11219185
anti-CD45RA	PerCP	1/100	HI100	AB_2616996
anti-CD95	BV650	1/100	DX2	AB_2629739
anti-CD107a	BV711	1/200	H4A3	AB_2565839
anti-CD127	BUV737	1/20	HIL-7R-M21	AB_2870121
anti-CD185	AF488	1/100	J252D4	AB_2561893
anti-CD197	APC	1/100	G043H7	AB_10915474
anti-CD279	DE-Dazzle 594	1/20	EH12.2H7	AB_2563658
anti-Foxp3	PE	1/20	206D	AB_492987
anti-Ki-67	PE-Cy7	1/1000	Ki-67	AB_2562871

antibody	conjugate/dye	dilution	clone	RRID
viability	blue fluorescent reactive dye	1/1000		
anti-CD14	BV421	1/100	63D3	AB_2810580
anti-CD19	Alexa Fluor 700	1/50	HIB19	AB_493751
anti-CD3	BV510	1/100	UCHT1	AB_2563468
anti-CD4	FITC	1/100	SK3	AB_1953236
anti-CD8a	APC/Fire750	1/100	RPA-T8	AB_2572095
anti-CD137	PE-Dazzle	1/50	4B4-1	AB_2566260
anti-CD154	PE-Cy7	1/50	24-31	AB_2563017
anti-CD137	PE-Dazzle	1/50	4B4-1	AB_2566260
anti-CD154	PE-Cy7	1/50	24-31	AB_2563017
anti-IFN-γ	PerCP-Cy5.5	1/50	4S.B3	AB_315234
anti-IL-2	APC	1/50	MQ1-17H12	AB_315097
anti-IL-4	PE	1/50	MP4-25D2	AB_2563879
anti-IL-17	BV786	1/28.5	BL168	AB_2566765

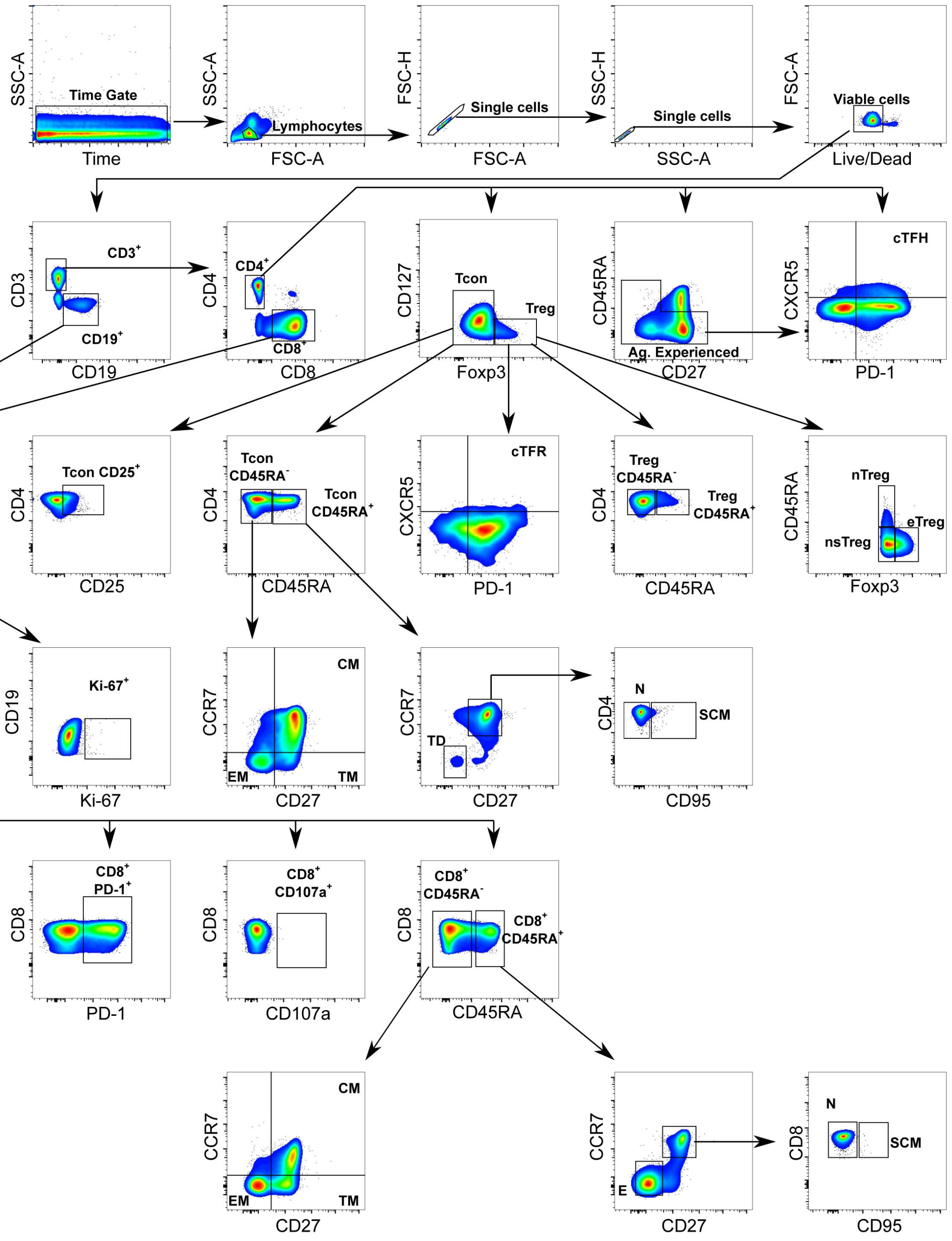

Supplementary Figure 1: Gating strategy for lymphocyte gating applicable to the flow cytometry results presented in Figure 2 and Supplementary Figures 3-5. The gating strategy was conducted in FlowJo™ on lymphocyte samples measured in a Cytoflex LX (Beckman Coulter).

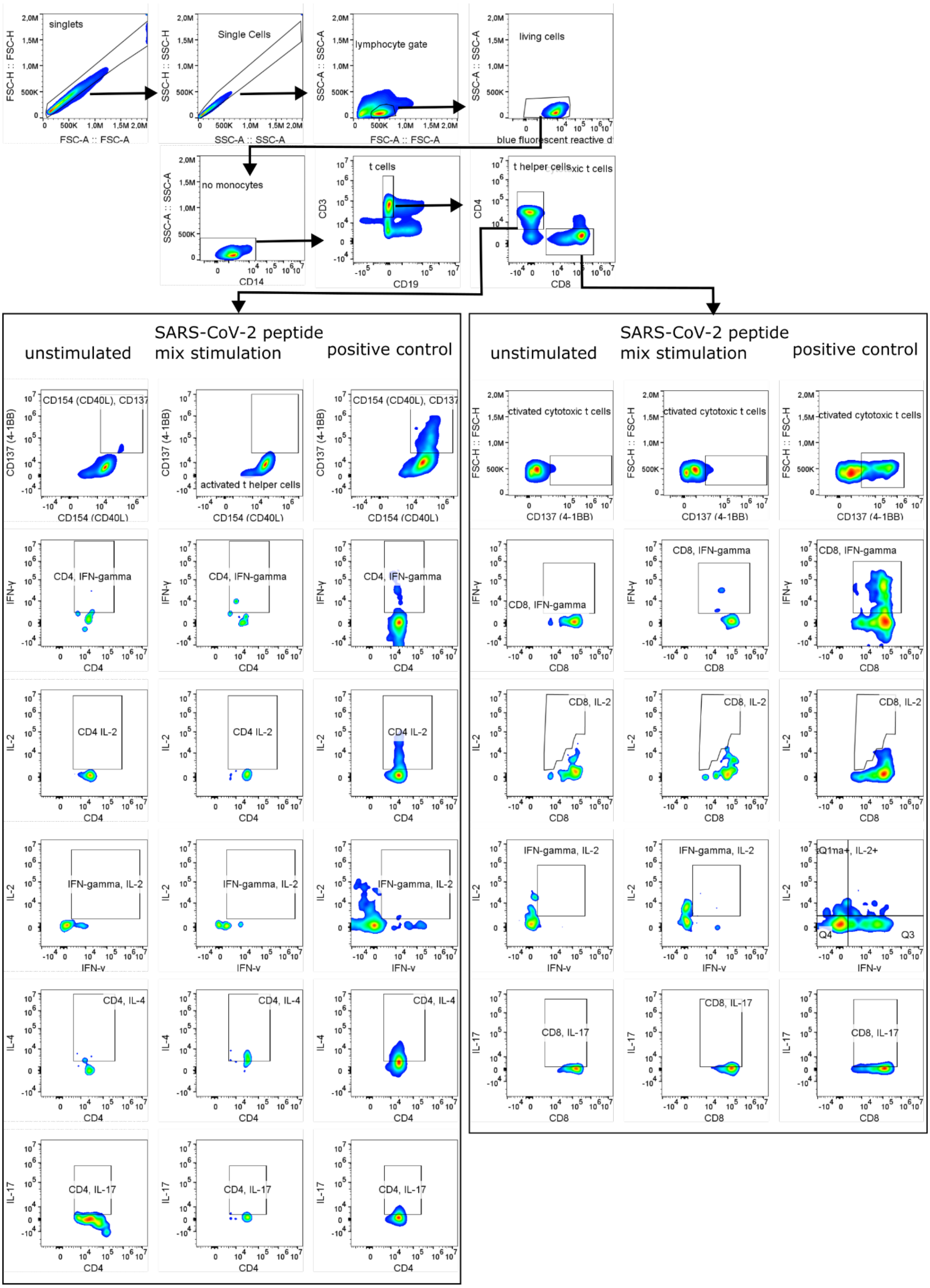

Supplementary Figure 2: Gating strategy for restimulated peripheral blood mononuclear cells . The gating strategy was used for the flow cytometry data presented in Fig. 3-5 and Fig. S6. Unstimulated controls and CytoStim™ stimulated positive controls were used for setting

the gates of positive cells in cytokine gating after SARS-CoV-2 peptide mix stimulation. For the comparisons of unstimulated, SARS-CoV-2-peptide mix and positive control, the same participant was used for the presented cytokine example pictures.

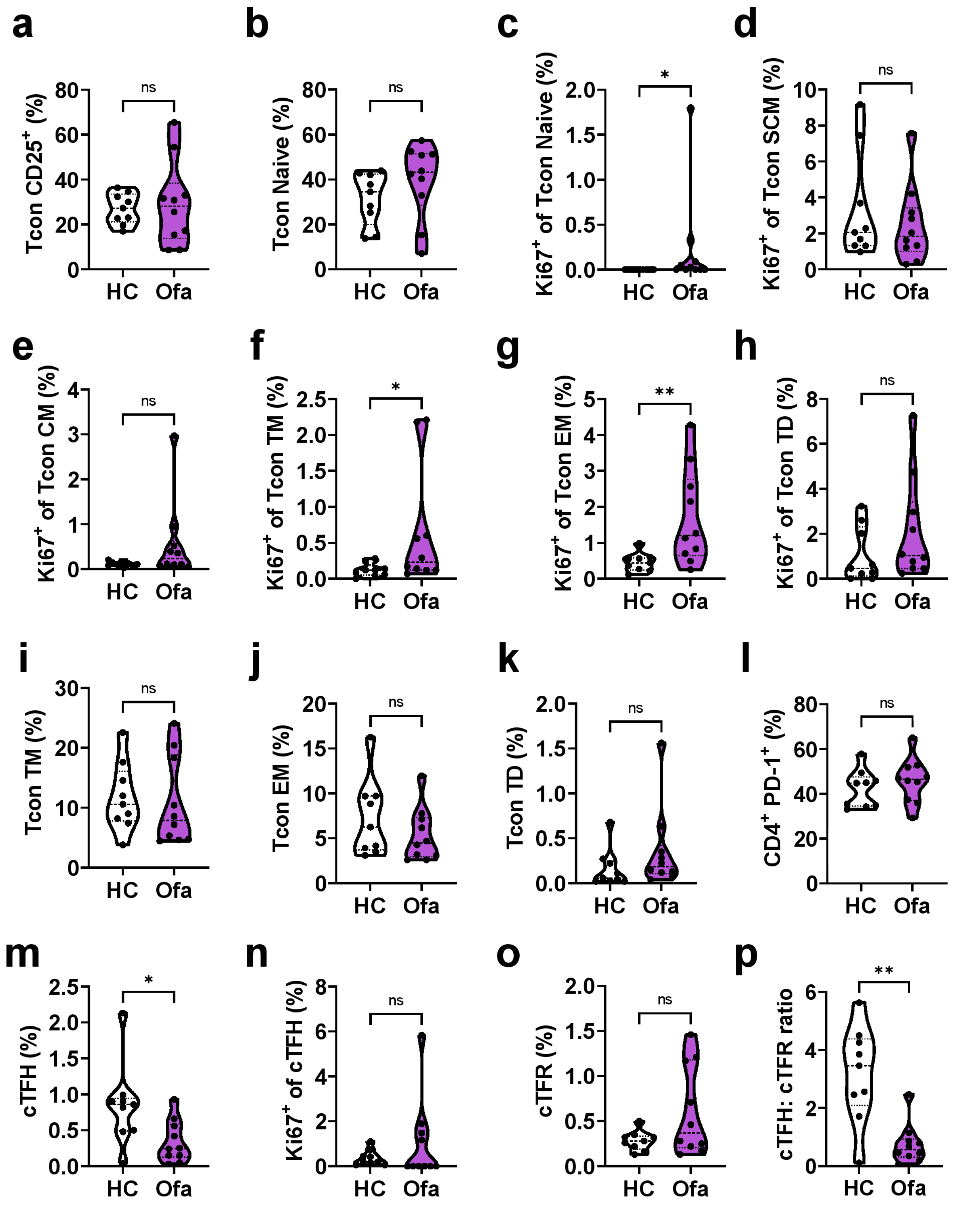

Supplementary Figure 3: Additional cell frequencies of the T helper cell phenotyping of MS patients under ofatumumab treatment and healthy controls. Immunophenotyping was conducted in ofatumumab treated multiple sclerosis patients (Ofa) and healthy controls (HC) using multi-color FACS. Subpopulations of $CD4^+$ T cells: conventional T cells (Tcon), stem cell like memory cells (SCM), central memory cells (CM), transitional memory cells (TM), effector memory cells (EM), terminally differentiated cells (TD), circulating T follicular helper

cells (cTFH), circulating T follicular regulatory cells (cTFR). Data were analyzed with a two-tailed Mann-Whitney test. n=10 Ofa, n=9 HC. *p<0.05, **p<0.01.

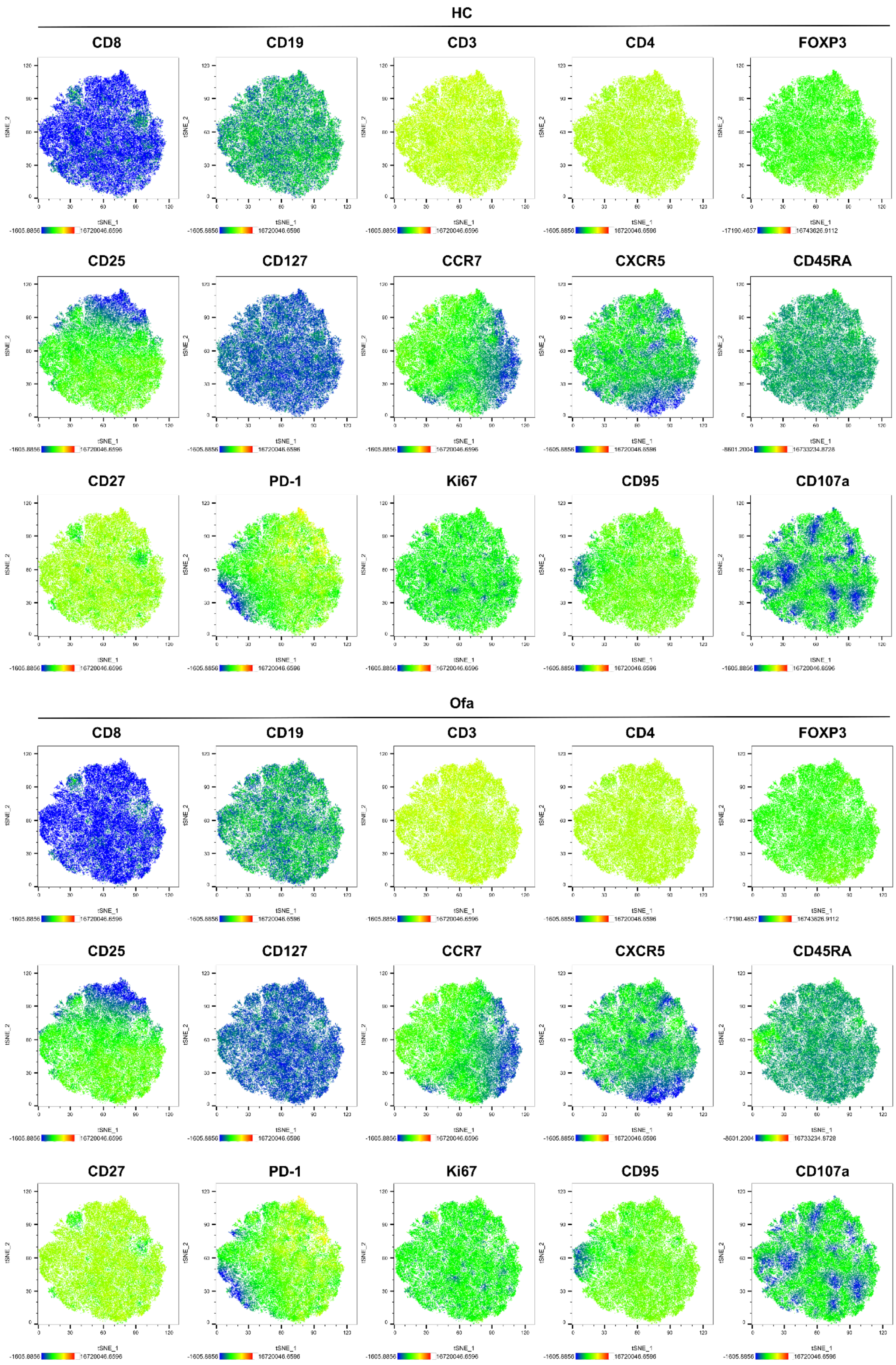

Supplementary Figure 4: Immunophenotyping of Treg cells. tSNE projection of the

indicated protein expression in MS patients under ofatumumab treatment and healthy controls.

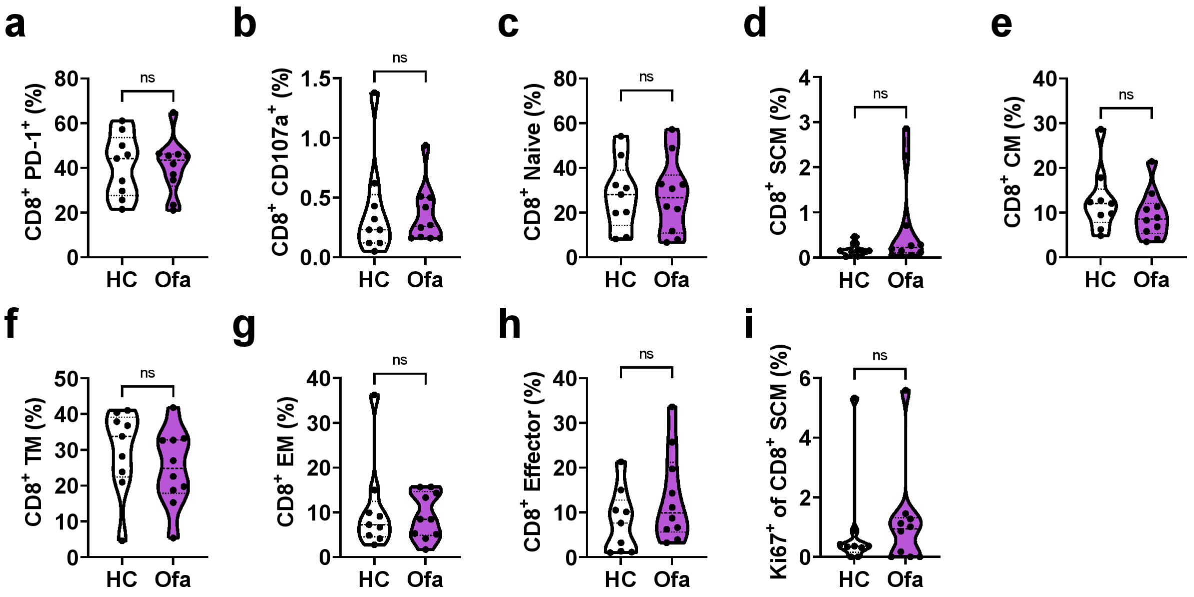

Supplementary Figure 5: Additional cytotoxic T cell frequencies of the lymphocyte phenotyping of MS patients under ofatumumab treatment and healthy controls. Immunophenotyping was conducted in Ofa and HC using multi-color FACS. Subpopulations of $CD8^+$ T cells: stem cell like memory cells (SCM), central memory cells (CM), transitional memory cells (TM), effector memory cells (EM). Data were analyzed with a two-tailed Mann-Whitney test. n=10 Ofa, n=9 HC.

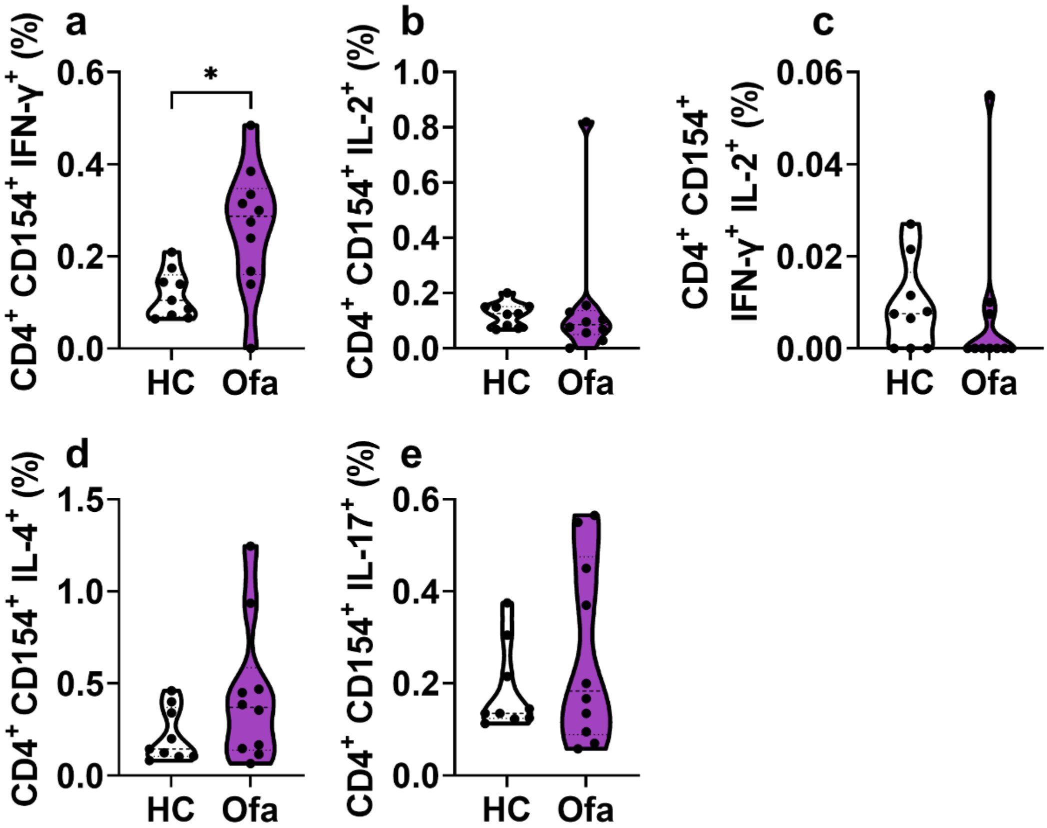

Supplementary Figure 6: Alternative analysis of activated T helper cells gating with only CD154 serving as activation marker. Peripheral blood mononuclear cells of Ofatumumab treated MS patients (Ofa) and healthy controls (HC) were collected 4-13 weeks following the third vaccination against SARS-CoV-2 and restimulated with a SARS-CoV-2 peptide pool following immunophenotyping via FACS. (a) Ofatumumab treated MS patients expressed significantly more IFN-γ in T helper cells (Th1) (p=0.0108). (b, c) Other Th1 expression patterns with IL-2 showed no significant differences between Ofa and HC. (d) T helper-2 cell specific expression patterns were not significantly altered between HC and Ofa (p=0.1823). (e) T helper 17 cells did not differ. Data were analyzed with a two-tailed Mann-Whitney test. n=10 Ofa, n=9 HC. *p<0.05.

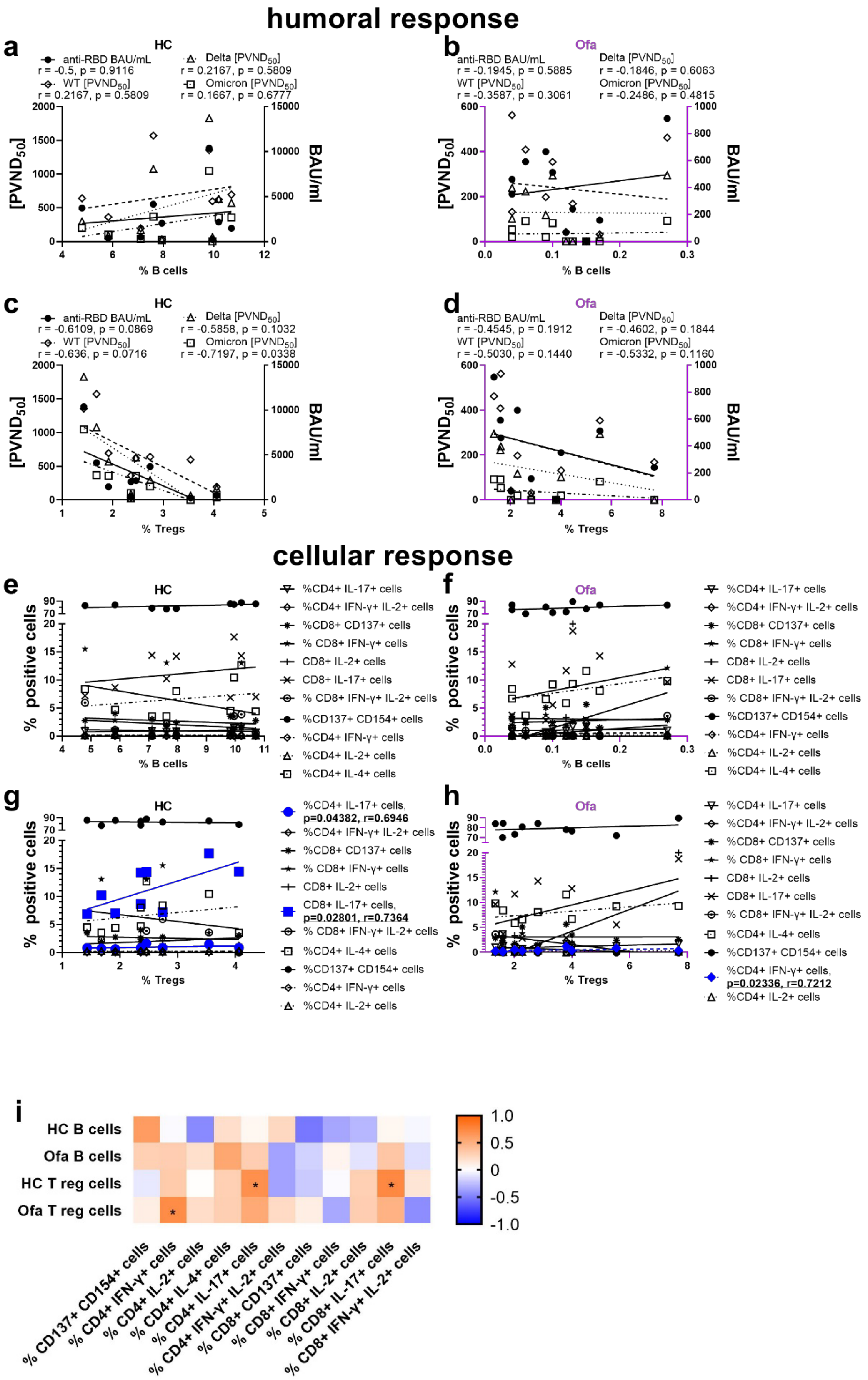

Supplementary Figure 7: Correlations of B cells and regulatory T cells with parameters of humoral and cellular immune response. a-d) Correlations of the humoral immune response depending on the frequency of B cells and Tregs prior to stimulation. (a, b) The anti SARS-

CoV-2 spike titer (BAU/mL) and the neutralization capacity of the serum [PVND50] did not correlate with B cell frequencies. (c) In HC, the neutralization capacity against Omicron was dependent on Treg frequencies. The other humoral response parameters were borderline significant for the correlation with %Tregs. (d) In the Ofa group, the humoral response showed a trend to a correlation with Tregs. (e-h) Correlations of the cellular immune response depending on the frequency of B cells and Tregs prior to stimulation. (e,f) Cellular immune response did not depend on the percentage of B cells present prior to stimulation in both HC and Ofa. (g) Frequencies of IL-17 expressing T helper and cytotoxic T cells correlated with the share of Tregs present before the stimulation in HC. (h) In Ofa patients, Th1 cells expressing CD4 and Interferon-γ correlated with Tregs. (i) Correlation matrix of B cells and regulatory T cells with cellular immune response, showing r values from panels e-h. Data were analyzed with Spearman correlation. n=10 Ofa, n=9 HC. *$p<0.05$.
